# Studies on mechanisms of interferon-gamma action in pancreatic cancer using a data-driven and model-based approach

**DOI:** 10.1186/1476-4598-10-13

**Published:** 2011-02-10

**Authors:** Falko Lange, Katja Rateitschak, Brit Fitzner, Ralf Pöhland, Olaf Wolkenhauer, Robert Jaster

**Affiliations:** 1 Department of Systems Biology and Bioinformatics, University of Rostock, 18051 Rostock, Germany; 2 Department of Medicine II, Division of Gastroenterology, Medical Faculty, University of Rostock, 18057 Rostock, Germany; 3 Unit of Reproductive Biology, Leibniz Institute for Farm Animal Biology, Wilhelm-Stahl-Allee 2, 18196 Dummerstorf, Germany

## Abstract

**Background:**

Interferon-gamma (IFNγ) is a multifunctional cytokine with antifibrotic and antiproliferative efficiency. We previously found that pancreatic stellate cells (PSC), the main effector cells in cancer-associated fibrosis, are targets of IFNγ action in the pancreas. Applying a combined experimental and computational approach, we have demonstrated a pivotal role of STAT1 in IFNγ signaling in PSC. Using *in vivo *and *in vitro *models of pancreatic cancer, we have now studied IFNγ effects on the tumor cells themselves. We hypothesize that IFNγ inhibits tumor progression through two mechanisms, reduction of fibrogenesis and antiproliferative effects on the tumor cells. To elucidate the molecular action of IFNγ, we have established a mathematical model of STAT1 activation and combined experimental studies with computer simulations.

**Results:**

In BALB/c-*nu/nu *mice, flank tumors composed of DSL-6A/C1 pancreatic cancer cells and PSC grew faster than pure DSL-6A/C1 cell tumors. IFNγ inhibited the growth of both types of tumors to a similar degree. Since the stroma reaction typically reduces the efficiency of therapeutic agents, these data suggested that IFNγ may retain its antitumor efficiency in PSC-containing tumors by targeting the stellate cells. Studies with cocultures of DSL-6A/C1 cells and PSC revealed a modest antiproliferative effect of IFNγ under serum-free conditions. Immunoblot analysis of STAT1 phosphorylation and confocal microscopy studies on the nuclear translocation of STAT1 in DSL-6A/C1 cells suggested that IFNγ-induced activation of the transcription factor was weaker than in PSC. The mathematical model not only reproduced the experimental data, but also underscored the conclusions drawn from the experiments by indicating that a maximum of 1/500 of total STAT1 is located as phosphorylated STAT1 in the nucleus upon IFNγ treatment of the tumor cells.

**Conclusions:**

IFNγ is equally effective in DSL-6A/C1 tumors with and without stellate cells. While its action in the presence of PSC may be explained by inhibition of fibrogenesis, its efficiency in PSC-free tumors is unlikely to be caused by direct effects on the tumor cells alone but may involve inhibitory effects on local stroma cells as well. To gain further insights, we also plan to apply computer simulations to the analysis of tumor growth *in vivo*.

## Background

Accumulation of somatic mutations in genes regulating cell growth, survival and differentiation represents a key mechanism in the process of molecular carcinogenesis. Over the last two decades, a large number of oncogenic and anti-oncogenic signal transduction networks have been described and generated considerable interest in novel approaches aimed at a more systematic understanding of interactions between cellular proteins involved in tumorigenesis. One strategy to meet this goal is the introduction of mathematical models into basic oncological research, taking advantage of recent progress in the field of systems biology [1]. Most importantly, computational models are not only descriptive but may also be used to make predictions that can be tested experimentally. The growing value of systems biology approaches in molecular research has raised hopes that mathematical models may also be applied to clinical oncology. To this day, however, there remains a considerable gap between the level of modeling (mostly related to subcellular processes) and the complexity of tumor growth *in vivo*.

Here, we describe a pilot study designed to gain molecular insights into the action of an antineoplastic agent in pancreatic cancer (PC), a devastating disease with the worst prognosis of all common human tumors [2,3]. One of the key features of PC is the extended fibrosis, which has been linked to the activation of pancreatic stellate cells (PSC) [4,5]. The desmoplastic reaction not only accompanies the disease, but plays an active role in its progression and reduces the efficiency of cytostatic drugs [5-8]. We have previously shown that interferon-γ (IFNγ) inhibits fibrogenesis by targeting PSC, and established an ODE (ordinary differential equation)-model of IFNγ signaling through STAT1 (signal transducer and activator of transcription) in PSC [9,10]. Simulations of the parametric model not only reproduced experimental data well, but also generated predictions regarding the activation of the STAT1 pathway that could be experimentally verified.

Interferons are multifunctional cytokines with antiviral, antiproliferative and immunomodulatory effects [11]. Their antineoplastic action is exploited in the treatment of several human malignancies. Thus, IFNα has been proposed as an active component for the treatment of PC as part of a chemoradiation protocol [12,13]. It is currently unknown, however, which cells represent the main targets of IFN action *in vivo *(PC cells, PSC, other cells?), and what are the molecular determinants of IFN efficiency or inefficiency.

Using a mouse model of heterotopic PC, we initially studied how IFNγ affects the growth of tumor cells in the presence and absence of co-injected PSC. Accompanying *in vitro *studies were performed to determine the growth-inhibitory effect of the cytokine on DSL-6A/C1 pancreatic cancer cells and cocultures of tumor cells with PSC. Furthermore, we analyzed IFNγ signaling through STAT1 in DSL-6A/C1 cells in a quantitative manner. Subsequently, the signaling data were integrated into a mathematical model of IFNγ action in DSL-6A/C1 cells, and linked to the results of our previous studies with stellate cells. Experimental and computational data together suggest that the tumor cells are less responsive to IFNγ in that they require higher doses of the cytokine for efficient STAT1 activation and growth inhibition. Interestingly, IFNγ nevertheless inhibited the growth of PSC-free tumors *in vivo*, suggesting the existence of additional cellular targets, such as local stroma cells.

## Results

### Effects of IFNγ on PC growth *in vivo *and *in vitro*

**Figure 1:**
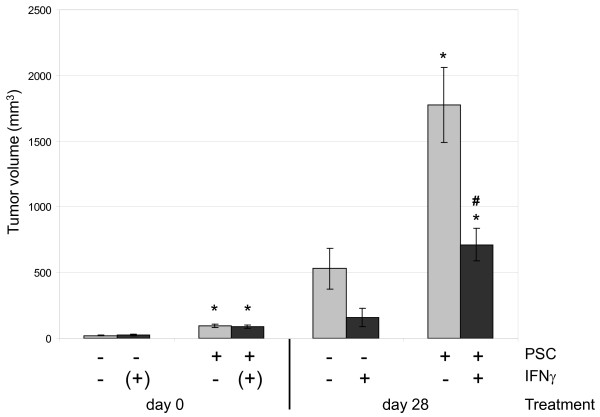
**Growth rates of PSC-containing and PSC-free DSL-6A/C1 tumors in nude mice and effects of IFNγ**. Injections of tumor cells, alone or together with PSC, was performed as described in the "Methods" section. Treatment with IFNγ (10 μg per day for 4 weeks, n = 4) started when the faster-growing tumor had reached a size of 6 mm in one dimension (day 0). Initiation of IFNγ treatment is indicated by the symbol (+). Control animals (n = 6) received solvent injections only. Indicated are the average values of the tumor volumes ± SEM. * p < 0.05 vs. without PSC (paired t-test), # p < 0.05 vs. untreated (unpaired t-test).

**Figure 2:**
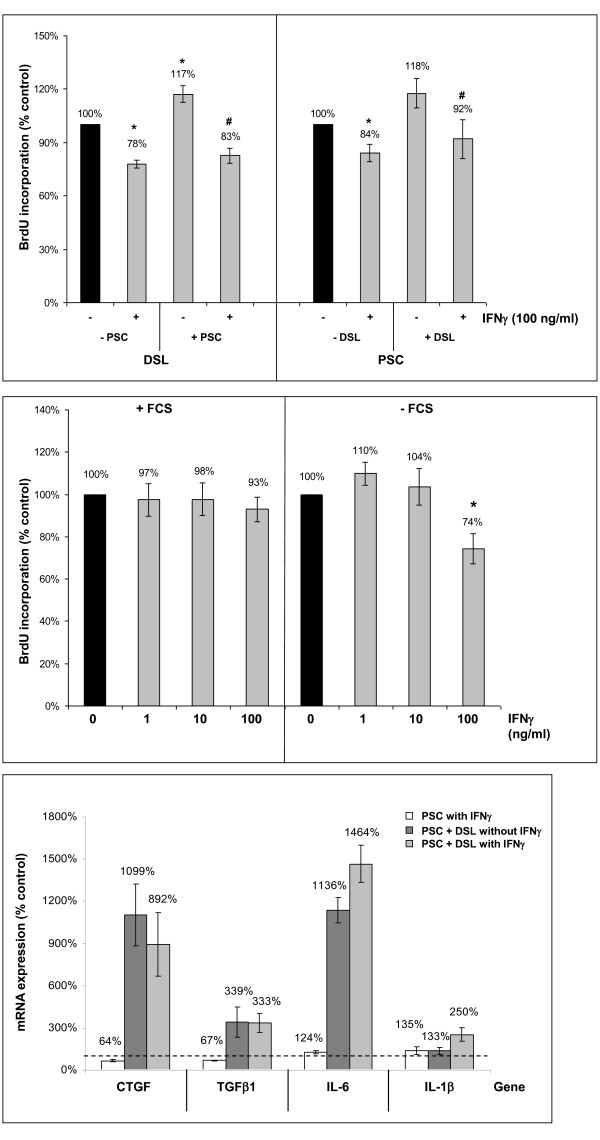
**Effects of IFNγ on the DNA synthesis and gene expression**. Upper panel: DSL-6A/C1 cells and PSC were either cultured alone (columns 1, 2, 5 and 6) or together (columns 3, 4, 7 and 8) in transwell plates. After IFNγ treatment for 48 h under serum-free conditions as indicated, proliferation of DSL-6A/C1 cells (columns 1-4) and PSC (columns 5-8) was assessed with the BrdU incorporation assay. One hundred percent cell proliferation corresponds to DSL-6A/C1 cells or PSC cultured alone and without IFNγ. Data from 12 separate cultures were used to calculate mean values and SEM. * p < 0.05 vs. untreated monocultures, # p < 0.05 versus untreated cocultures (Wilcoxon's rank sum test). Middle panel: DSL-6A/C1 cells were cultured with or without FCS and IFNγ as indicated for 24 h, before DNA synthesis was assessed with the BrdU incorporation assay. One hundred percent BrdU incorporation corresponds to controls cultured without IFNγ. Data are presented as mean ± SEM (n = 6 separate cultures); * p < 0.05 vs. control cultures (Wilcoxon's rank sum test). Lower panel: Monocultures of PSC and cocultures of PSC with DSL-6A/C1 cells were treated with IFNγ at 100 ng/ml for 24 h. The mRNA expression of *CTGF*, *TGF-β1*, *IL-6*, *IL-1β *and the housekeeping gene *HPRT *in PSC was analyzed by real-time PCR, and relative amounts of target mRNA were calculated as described in the "Methods" section. One hundred percent mRNA expression of each gene (dotted line) corresponds to PSC monocultures grown without IFNγ. Data of 3 independent experiments (with triplicate samples) were used to calculate mean values and SEM.

To test the antitumor and antifibrotic efficiency of IFNγ *in vivo*, we established a mouse model of PC in which the effects of the drug could be directly related to the presence or absence of activated stellate cells. Therefore, BALB/c-*nu/nu *mice (subsequently termed nude mice) were injected with either DSL-6A/C1 pancreatic cancer cells alone (one side), or a mixture of DSL-6A/C1 cells and PSC (opposite flank). Systemic treatment with IFNγ was initiated when the faster-growing tumor had reached a size of 6 mm in one dimension, and continued for 28 days. In accordance with published data [6], tumors grew faster if PSC were co-injected (Figure 1). As indicated by the tumor volumes, IFNγ-treatment diminished the growth rate of pure DSL-6A/C1 tumors in a non-significant manner (columns 5 and 6). On PSC-containing tumors, IFNγ displayed a quantitatively similar, but statistically significant inhibitory effect (columns 7 and 8). Based on these data, we hypothesized that both stellate and cancer cells may be targets of antiproliferative effects of IFNγ. Expanding previous studies with cultured PSC, we therefore investigated the antiproliferative effects of IFNγ in a coculture model of DSL-6A/C1 cells and PSC. In agreement with previous studies [14], PSC stimulated the growth of the tumor cells and vice versa (Figure 2 upper panel), although the effect of stellate cells on DSL-6A/C1 cells just missed statistical significance (column 7; p = 0.06). IFNγ at 100 ng/ml significantly inhibited the growth of both types of cells under monoculture and coculture conditions, providing evidence for the hypothesis described above. However, the inhibitory effect of the cytokine on the growth of DSL-6A/C1 cells was lost if the experiment was performed in the presence of serum, or IFNγ concentration reduced to ≤ 10 ng/ml (Figure 2 middle panel). In contrast, we have previously shown that proliferation of PSC was significantly reduced by IFNγ even in the presence of FCS, and at a concentration as low as 1 ng/ml [9]. In order to gain mechanistic insights into the cellular interactions, we analyzed the effect of the coculture on PSC gene expression, focusing on a panel of established PSC-derived mediators. As shown in Figure 2 (lower panel), expression of connective tissue growth factor (CTGF), transforming growth factor-β1 (TGF-β1) and interleukin (IL)-6 (but not IL-1) in PSC was stimulated by cocultured tumor cells. The effects of IFNγ on PSC gene expression were altogether mild, with a roughly two-fold up-regulation of IL-1β in cocultures as the most pronounced phenomenon.

### STAT1 pathway activation in DSL-6A/C1 cells: biological data and mathematical model

**Figure 3:**
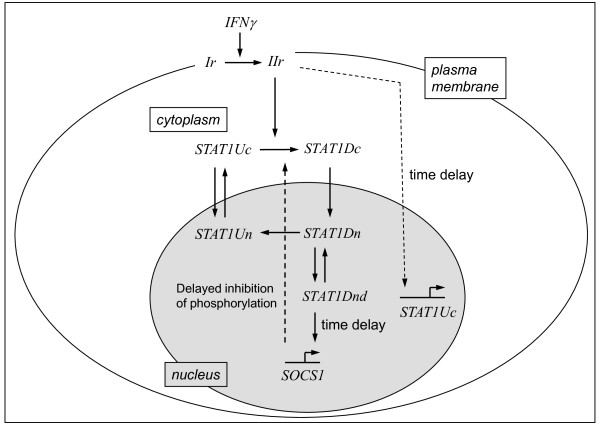
**Reaction network of the IFNγ stimulated STAT1 signaling pathway**. The network shows key reactions of the pathway, which are translated into a mathematical model. IFNγ activates the receptor Ir. To keep the model simple, Janus kinases are not considered separately but as part of the active receptor complex IIr only. IIr phosphorylates unphosphorylated cytosolic STAT1 (STAT1Uc), followed by rapid formation of homodimers (STAT1Dc). STAT1Dc translocates into the nucleus (STAT1Dn) and induces the transcription of specific target genes by binding to the DNA (STAT1Dnd). When dimerized nuclear STAT1 is not bound to the DNA, dimers may dissociate, followed by protein dephosphorylation and nuclear export of the resulting STAT1Un [27]. STAT1Uc can also shuttle into the nucleus [32]. The network considers *SOCS1 *as a potential negative feedback regulator inhibiting the phosphorylation of STAT1Uc [28,29] and *STAT1 *itself as target genes of IFNγ-activated signaling [10]. The annotation *time delay *refers to temporal differences between IFNγ action at the receptor level and consecutive steps. The level of phosphorylated STAT1 did not decrease despite induction of *SOCS1 *expression (Fig. 5). This observation suggests that the negative feedback could be effective at late times (≥ 180 min) only, where it could reduce the slope of the late increase of phosphorylated STAT1. In comparison to the network previously established for stellate cells [10], only two reactions were changed: Since the experimental data showed no decrease of STAT1 phosphorylation, IFN**γ **degradation and receptor deactivation were neglected.

The differences in the biological responsiveness of PSC and DSL-6A/C1 cells to IFNγ prompted us to ask if they could be linked to different efficiencies of the cytokine in the activation of the key transcription factor in IFNγ signaling, STAT1. To address the question, we combined experimental analysis of STAT1 pathway activation in DSL-6A/C1 cells with computer simulations, taking advantage of a previously established mathematical model of STAT1 signaling in PSC [10]. The reaction network used for computational analysis (Figure 3) contained slight modifications of the previous one [10], which are specified in the figure legend. Subsequently, the network was translated into a system of ODEs, describing temporal changes of the network components as a function of interactions and transport processes. The parameters of the model include reaction constants, delay times and the total receptor concentration. Parameter estimation was done by global optimization from protein and mRNA time series for two different concentrations of IFNγ (10 and 100 ng/ml). The ODEs and the optimized parameter values are provided in the Additional files 1 and 2.

**Figure 4:**
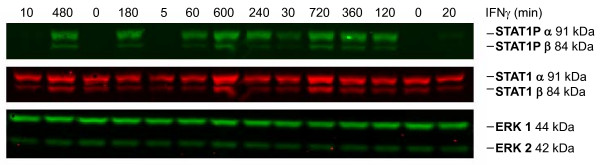
**Time course of phospho-STAT1 and STAT1 protein levels in IFNγ-stimulated DSL-6A/C1 cells**. The cells were stimulated under serum-free conditions with IFNγ (100 ng/ml) for up to 720 min. Total cellular lysates from an equal number of cells were subjected to immunoblot analysis. Primary antibodies were detected with the help of fluorescein (IRDye^®^)-labeled secondary antibodies. Phospho-STAT1 (STAT1P), STAT1 protein and ERK 1/2 staining are exemplarily shown for one blot. The samples were loaded in a randomized order to exclude lane correlated blotting errors caused by gel and transfer inhomogeneities, as previously suggested [33].

**Figure 5:**
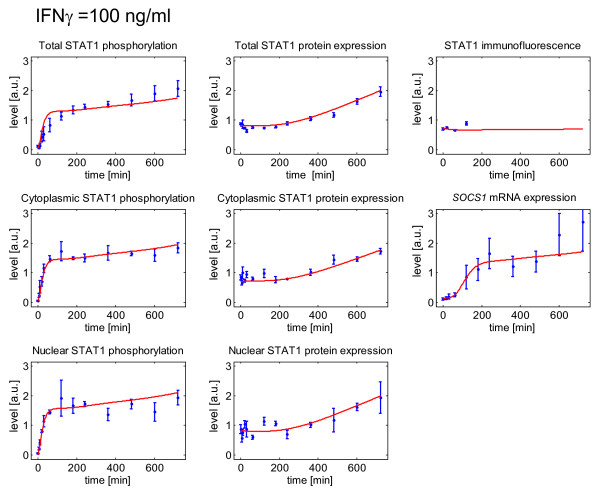
**IFNγ-induced STAT1 pathway activation: Experimental time series and computational simulation results**. *Experimental data: *DSL-6A/C1 cells were stimulated with IFNγ at 100 ng/ml for the indicated periods of time. Experimentally determined levels of phospho-STAT1, total STAT1 protein (immunoblot data received from total cellular lysates, the cytosolic cell fraction and the nuclei, respectively) and *SOCS1 *mRNA were normalized as described in the "Methods section". They are expressed in arbitrary units (a.u.) as averaged values of four to six independent experiments (± SEM). The immunoblot and PCR time series were scaled according to the requirements of the optimization methods. Confocal microscopy data were processed by calculating the ratio of nuclear versus cytoplasmic STAT1 concentration. The data are averaged values (± SEM) of 30 to 40 cells from three to four different images for each time point. Measured data are presented by blue symbols with error bars. *Computational simulations: *The simulated time courses resulting from the mathematical model with optimized parameters for STAT1, nuclear translocation of STAT1, STAT1P and *SOCS1 *mRNA are presented by solid red lines.

As shown in Figure 4 IFNγ at the high dose of 100 ng/ml rapidly induced tyrosine phosphorylation of STAT1. Both, a conventional visualization of the experimental data and the application of the mathematical model concordantly suggested that the increase of phospho-STAT1 happened in two phases: An initial phase of rapid phosphorylation was followed by a second phase characterized by a slow but long-lasting further increase of phospho-STAT1 (Figure 5 upper left diagram). This second phase corresponded to a rise of STAT1 protein levels (upper middle diagram). Increased levels of phospho-STAT1 and STAT1 protein were synchronously detectable in the cytosolic and nuclear cell fraction (middle and lower row, respectively), and well reproduced by the mathematical model. Accordingly, the STAT1 target gene *suppressor of cytokine signaling *(*SOCS1*) also displayed a biphasic pattern of enhanced expression (middle row, right diagram).

**Figure 6:**
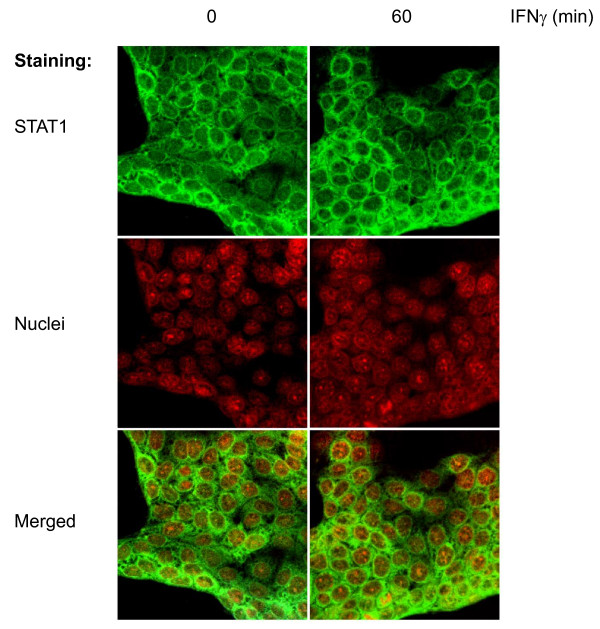
**Immunocytochemical analysis of total STAT1 expression and subcellular localisation**. DSL-6A/C1 cells treated with IFNγ (100 ng/ml) as indicated were stained by incubation with specific antibodies detecting STAT1. Upper row: Binding of primary antibody was visualized with an Alexa Fluor^® ^488-labeled secondary antibody, resulting in green fluorescence. Middle row: Nuclei were counterstained with TO-PRO^®^-3 iodide (shown in red color). Lower row: merged pictures. Microphotographs were analyzed by confocal laser scanning microscopy (original magnification × 400).

A quantitative analysis of confocal microscopy data (see Figure 6 for a typical example) and computational simulation together (Figure 5 upper row, right diagram) revealed the surprising finding that IFNγ-induced phosphorylation of STAT1 left the ratio of nuclear to cytoplasmic STAT1 almost unchanged. We therefore hypothesize that only a small fraction of cytoplasmic STAT1 was activated, and translocated to the nucleus, after IFNγ stimulation. Direct evidence in support of this hypothesis comes from parameter estimation of the mathematical model: According to these results a relative concentration of 1/500 of total STAT1 is an upper boundary for the concentration of phosphorylated STAT1 in the nucleus. This is presented in detail in the Additional file 3.

**Figure 7:**
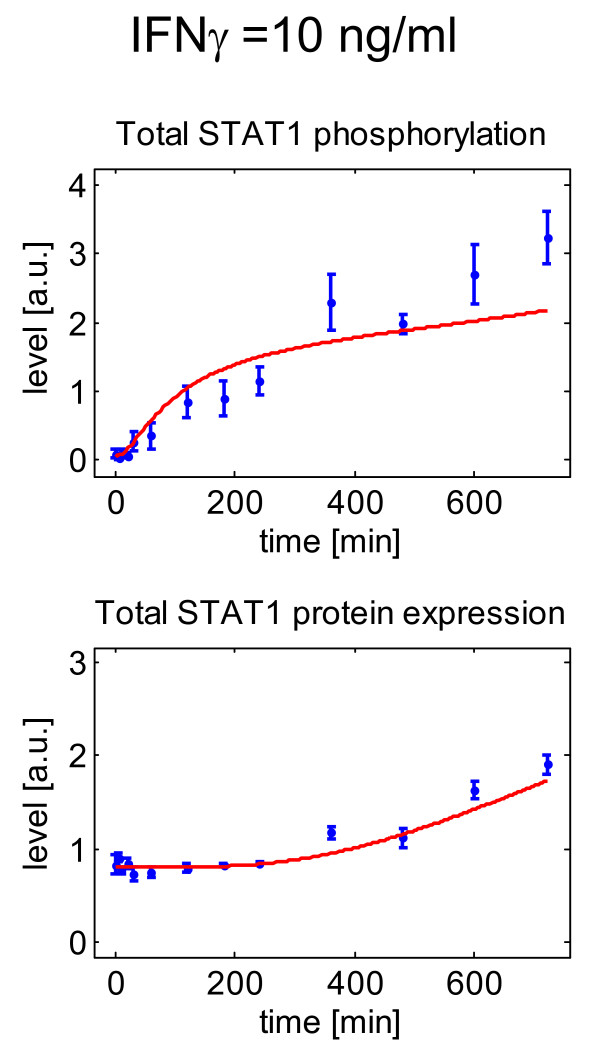
**Effects of IFNγ at 10 ng/ml on STAT1 protein expression and phosphorylation**. *Experimental data: *DSL-6A/C1 cells were stimulated with IFNγ for the indicated periods of time. Phospho-STAT1 and STAT1 protein levels in total cellular lysates were determined by immunoblot analysis. The data were processed as described in the legend to Fig. 5. Averaged values (± SEM) of four independent experiments are presented by blue symbols. *Computational simulations: *The simulated time courses resulting from the mathematical model are presented by solid red lines.

We also tested the effects of a lower dose of IFNγ, 10 ng/ml, on STAT1 activation (Figure 7). Both, visualization of experimental data and computational simulations of the mathematical model showed that dose reduction resulted in a slower rise of phospho-STAT1 levels (upper panel), while the enhanced expression of STAT1 protein at later time points after IFNγ application was retained (lower panel). At 1 ng/ml, IFNγ did not activate STAT1 at all (data not shown). Together, these results suggest that the antiproliferative effect of IFNγ in DSL-6A/C1 cells is closely related to the activation of STAT1.

## Discussion

Integrating the design of experiments with mathematical modeling is considered a promising approach to unravel the molecular complexity of tumors [1]. Although computational models may be applied to various fields of cancer research, an improved mechanistic understanding of relevant molecular processes represents a key goal, at least at the current stage. Here, we have analyzed the biological and molecular effects of IFNγ in rat pancreatic cancer cells, using a combined experimental and computational approach. Our studies were motivated by the following considerations:

Interferons are of clinical interest in the treatment of pancreatic cancer [12,13]. Moreover, they display antifibrotic effects in different tissues [9,15,16], suggesting that they may enhance direct antitumor action by inhibiting pancreatic fibrosis; a progression factor of the disease. We have previously shown that pancreatic stellate cells, the main effector cells in fibrosis, are highly susceptible to IFNγ treatment [9], and developed a mathematical model of IFNγ signaling through STAT1 [10], the principal transcription factor in the mediation of IFNγ effects [17,18]. By expanding experimental and modeling efforts to tumor cells, we expected new insights regarding the relative sensitivity of both cell types to the drug.

**Figure 8:**
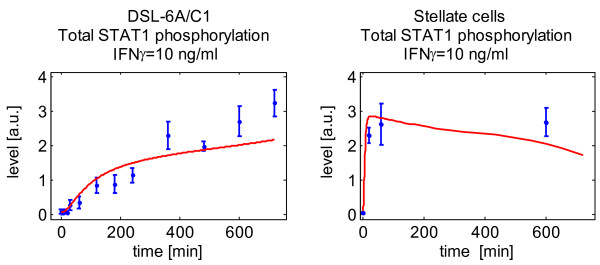
**Phosphorylation profiles of total STAT1 in DSL-6A/C1 cells and stellate cells treated with IFNγ at 10 ng/ml**. Shown are the data and computational simulations of this investigation (DSL-6A/C1 cells) and our previous study with stellate cells [10].

In contrast to stellate cells [9,19], DSL-6A/C1 cells did not respond to IFNγ by diminished cell proliferation as long as they were cultured in the presence of serum. A reduced rate of DNA synthesis was only observed when IFNγ was applied at the high dose of 100 ng/ml, and combined with another stressor, the withdrawal of serum. To obtain a detectable STAT1 phosphorylation in DSL-6A/C1 cells, an IFNγ concentration ≥ 10 ng/ml was required, while in cultured stellate cells 1 ng/ml was sufficient [10]. Furthermore, the activation profile of STAT1 in DSL-6A/C1 cells differed from the one previously observed in stellate cells in that a reduction of cytokine concentrations from 100 to 10 ng/ml was associated with a slower increase of phospho-STAT1 (Figure 8). As revealed by parameter value optimization of the mathematical model, a maximum of 1/500 of total STAT1 is located as phosphorylated STAT1 in the nucleus upon IFNγ treatment of DSL-6A/C1 cells. Together, these results indicate that the cancer cells are less sensitive towards IFNγ than pancreatic stellate cells, and suggest a less efficient activation of STAT1 signaling as a possible molecular explanation.

In the nude mouse model, it was found that PSC-containing tumors grew faster and to larger volumes than pure DSL-6A/C1 tumors. This finding is in agreement with previous studies, which have revealed that co-injected PSC accelerate the growth of pancreatic cancer in animal models of the disease [6,20,21]. Moreover, the tumor stroma in pancreatic cancer has been shown to induce resistance of the tumor cells to drug treatment [7,8,22]. Possible mechanisms include paracrine effects of the stroma cells, an inefficient delivery of the antineoplastic agent to the tumor tissue, and a protection of the tumor against the immune system of the host (less relevant in the immunodeficient nude mouse model). In this context, it was interesting to note that IFNγ inhibited the growth of tumors with and without stellate cells in a similar manner.

When grown in coculture, both PSC and DSL-6A/C1 cells proliferated at a higher rate than monocultures of the respective cell type, confirming previous results [14]. The antiproliferative effect of IFNγ on both types of cells, however, remained significant, suggesting that its efficiency was not affected by the paracrine interactions between tumor and stellate cells. Molecular coupling factors between PSC and DSL-6A/C1 factors may include CTGF, TGF-β1 and IL-6, all of which were found to be up-regulated in PSC upon coculture, and are known to be involved in PSC activation [5]. In the coculture model, IFNγ increased expression of IL-1β, which might favor the development of a pro-inflammatory microenvironment in the tumor.

The sensitivity of pure DSL-6A/C1 tumors towards IFNγ treatment warrants further investigation. In pilot studies, we found that DSL-6A/C1 cells can attract local fibroblasts to the tumor, where they may exert effects of tumor stroma cells and are possibly targeted by IFNγ.

It has to be noted that the antitumor efficiency of IFNγ may also depend on the differentiation status of the pancreatic cancer cells: IFNγ-responsive DSL-6A/C1 tumors are relatively well differentiated. Purely differentiated tumors of Panc02 cells [22], however, were found to be resistant to IFNγ treatment when growing in C57Bl/6N mice (R. Jaster, unpublished data).

To this end, we have used the systems biology approach for the *in vitro *part of our experiments only. Nevertheless, modeling and simulation proved helpful since it provided additional mechanistic insights into the action of IFNγ, which was the focus of this study. We are currently extending the effort to the simulation of experimental tumor growth *in vivo*. We believe that in the long run modeling may also become a valuable tool to monitor and predict the efficiency of antineoplastic agents. A major challenge on this way is the lack of quantitative and kinetic data of high density and quality, appropriate for data-driven modeling in which parameter values of kinetic (dynamical) models are directly estimated from time course data.

## Conclusions

In a heterotopic nude mouse model of pancreatic cancer, IFNγ significantly inhibited the growth of tumors containing co-transplanted stellate cells, which accelerated tumor progression. On pure DSL-6A/C1 tumors, IFNγ displayed a quantitatively similar but statistically not significant growth inhibitory effect. PSC are more sensitive to the antiproliferative action of IFNγ than DSL-6A/C1 pancreatic cancer cells. A possible explanation is provided by our experimental data and computer simulations, since they concordantly indicate that in DSL-6A/C1 cells only a small fraction of the key transcription factor STAT1 is located as phosphorylated protein in the nucleus upon IFNγ stimulation. We therefore hypothesize that the antiproliferative effect of IFNγ on pure DSL-6A/C1 tumors is mediated, at least in part, by the targeting of additional cell types, such as local fibroblasts.

## Methods

### Materials

Iscove's modified Dulbecco's medium (IMDM), Dulbecco's Modified Eagle Medium (DMEM) and supplements for cell culture were obtained from Biochrom (Berlin, Germany), recombinant rat IFNγ from Immunotools (Friesoythe, Germany), and bovine serum albumin (BSA) from Roche Diagnostics (Mannheim, Germany). TriReagent^® ^was purchased from Ambion (Austin, TX, USA). Reverse transcription and Taqman^™ ^reagents were delivered by Applied Biosystems (Foster City, CA, USA). Primary antibodies were from BD Biosciences (Franklin Lakes, NJ, USA; anti-pY701-STAT1; mouse monoclonal), Upstate Cell Signaling Solutions (Billerica, MA, USA; anti-extracellular signal-regulated kinase (ERK) 1/2; rabbit polyclonal), Santa Cruz (Santa Cruz, CA, USA; anti-STAT1; rabbit polyclonal) and New England BioLabs (Frankfurt, Germany; anti-GAPDH; rabbit monoclonal and anti-lamin A/C; rabbit polyclonal). Fluorescently-labeled secondary antibodies for immunoblot analysis and Odyssey^® ^blocking buffer were supplied by LI-COR (Lincoln, NE, USA). Polyvinylidene fluoride (PVDF) membrane was from Millipore (Billerica, MA, USA), and TO-PRO^®^-3 iodide as well as Alexa Fluor^® ^reagents from Invitrogen (Darmstadt, Germany). Tissue culture dishes (corning plasticware) and all other chemicals were from Sigma (Deisenhofen, Germany).

### Cell culture

Stellate cells from rat pancreas were isolated by collagenase digestion of the organ followed by Nycodenz density gradient centrifugation as previously described [23], and cultured in IMDM supplemented with 17% fetal calf serum (FCS), 1% non-essential amino acids (dilution of a 100 × stock solution), 100 U/ml penicillin and 100 μg/ml streptomycin. PSC were harvested by trypsinization on day seven after isolation and recultured at equal seeding densities according to the experimental requirements. All experiments were performed with cells passaged no more than 2 times. The rat pancreas carcinoma cell line DSL-6A/C1 was obtained from American Type Culture Collection (ATCC), and cultured in DMEM supplemented with 10% FCS and antibiotics (100 U/ml penicillin; 100 μg/ml streptomycin).

Coculture studies were performed in 24-transwell plates using inserts with a pore size of 0.4 μm. Therefore, DSL-6A/C1 cells and PSC were seeded into the upper or lower chamber of separate transwells, respectively. After an overnight incubation, culture medium was replenished, transwell chambers were assembled by transferring the inserts, and IFNγ was added according to the experimental design. All cells were grown at 37°C in a 5% CO_2 _humidified atmosphere.

### Animal tumor model and IFNγ treatment

BALB/c-*nu/nu *mice were purchased from Charles River Laboratories (Sulzfeld, Germany), kept under standard laboratory conditions and fed a rodent chow diet. All experiments were performed according to the guidelines of the local animal use and care committee.

Three month old male mice were injected subcutaneously into the left hind flank with 1×10^6 ^DSL-6A/C1 cells, and a mixture of 1×10^6 ^DSL-6A/C1 cells/1×10^6 ^PSC into the opposite site. Over the entire period of investigation, tumor growth was monitored twice a week, and volumes of outgrowing tumors were evaluated according to the following formula: width^2 ^× length × 0.52 [24]. Treatment was initiated when the faster-growing tumor had reached a size of 6 mm in one dimension (defined as day 0). Therefore, the mice were randomized into two groups, and treated with IFNγ (intraperitoneal injections of 10 μg per day, n = 4), or solvent only (controls; n = 6) for 28 days. Afterwards, the animals were sacrificed, and tumors from both flanks removed for further analysis.

### Cell proliferation assay

To assess proliferation of cells growing alone or in coculture, incorporation of 5-bromo-2'-deoxyuridine (BrdU) into newly synthesized DNA was quantified using the BrdU labeling and detection enzyme-linked immunosorbent assay kit (Roche Diagnostics). Therefore, the cells were incubated with IFNγ and BrdU as indicated, and BrdU uptake of cells growing in the lower chamber (PSC and DSL-6A/C1 cells, respectively) was measured according to the manufacturer's instructions. In studies with monocultures of DSL-6A/C1 or PSC, transwell inserts were omitted. For each experimental regime (coculture studies, dose-response experiments and studies on the effects of FCS), the times of IFNγ treatment were optimized in pilot investigations.

### Immunoblotting

To receive unfractionated total cellular protein, boiling lysis buffer (2% sodium dodecyl sulphate [SDS], 10% glycerol, 5 mM ethylenediaminetetraacetic acid [pH 8.0], 62.5 mM Tris-HCl [pH 6.8], 0.01% 3,3',5,5'-tetrabromophenolsulfonphthalein, 5% β-mercaptoethanol) was added directly to the cell monolayer. Nuclear extracts and the cytosolic fraction of the cells were prepared using the ProteoJET^™ ^cytoplasmic and nuclear protein extraction kit (Fermentas, St. Leon-Rot, Germany) according to the instructions of the manufacturer.

Cellular proteins received from equal numbers of cells were separated by 8% SDS-polyacrylamide gel electrophoresis and blotted onto PVDF membrane. Afterwards, membranes were blocked for one hour using Odyssey^® ^blocking buffer, before primary antibodies were added and incubation continued for another hour. Simultaneous detection of tyrosine-phosphorylated STAT1 and total STAT1 was performed as previously described [10]. Expression of ERK 1/2, GAPDH and lamin A/C was investigated by adding specific antibodies separately to the lower part of the membrane. IRDye^® ^800CW and IRDye^® ^680CW conjugated secondary antibodies were applied for detection of primary antibody binding.

Using an Odyssey^® ^Infrared Imaging System, all immunoblots were scanned at a wavelength of 700 nm for detecting IRDye^® ^680 labeled antibodies and at a wavelength of 800 nm for IRDye^® ^800CW conjugated antibodies. Signal intensities were quantified by means of the Odyssey^® ^software version 3.16. Signals obtained for phosphorylated STAT1 and total STAT1 protein were normalized for loading differences by calculating the ratio of phospho-STAT1 (or STAT1) to ERK 1/2 (total cellular protein), GAPDH (cytosolic fraction) and lamin A/C (nuclear extracts). Under the experimental conditions used in this study, protein levels of ERK 1/2, GAPDH and lamin A/C in the cells remained constant (data not shown). In a second step each time series was normalized to its average value over time. Finally the average value of four to six experiments was calculated for each time point.

### Confocal microscopy

DSL-6A/C1 cells were cultured on coverslips. After stimulation with IFNγ, the cells were fixed in methanol at -20°C for 10 min and washed twice with TBS (20 mM Tris, 150 mM NaCl, pH 7.6; supplemented with 0.5% BSA, 0.05% Tween20). Nonspecific binding sites were blocked with Roti^®^-ImmunoBlock solution (Carl Roth, Karlsruhe, Germany). Afterwards, the fixed cells were incubated with anti-STAT1 (in Roti^®^-ImmunoBlock solution) overnight at 4°C. After washing with TBS, the coverslips were stained with Alexa Fluor^® ^488 goat anti-rabbit IgG. Subsequently, the cells were washed and nuclei were stained with TO-PRO^®^-3 iodide. Finally, the cells were fixed with 2% paraformaldehyde for 15 min and embedded in mounting medium (25% glycerol, 10% polyvinylalcohol, 0.4% phenol, 0.05 M Tris, pH 8.5) on microscope slides. Fluorescence analysis was performed by using a confocal laser scanning microscope LSM 5 Pascal (Axiovert 200 M) and Zen 2007 (4.5) software (Carl-Zeiss, Jena, Germany). Images were further analyzed using the ImageJ (Open Source software package). To quantify nuclear translocation of STAT1, averaged ratios of nuclear versus cytoplasmic concentration (sum of pixel intensity divided by area) of STAT1 were calculated. Therefore, 30 to 40 cells from three to four different images were analyzed for each time point.

### Quantitative reverse transcriptase-PCR using real-time TaqMan^™ ^technology

DSL-6A/C1 cells and PSC, growing in FCS-containing culture medium, were treated with IFNγ as indicated, and total RNA was isolated with TriReagent^® ^according to the manufacturer's instructions. Subsequently, RNA was reverse transcribed into cDNA by means of TaqMan^™ ^Reverse Transcription Reagents and random hexamer priming. Target cDNA levels were analyzed by quantitative real-time PCR using TaqMan™ Universal PCR Master Mix and the following Assay-on-Demand^™ ^rat gene-specific fluorescently labelled TaqMan™ MGB probes in an ABI Prism 7000 sequence detection system (Applied Biosystems): Rn00595838_s1 (SOCS1), Rn00573960_g1 (CTGF), Rn00580432_m1 (IL-1β), Rn00561420_m1 (IL-6), Rn00572010_m1 (TGF-β1) and Rn01527840_m1 (hypoxanthine-guanine phosphoribosyl transferase [HPRT]). PCR was started with an initial denaturation at 95°C for 10 min, followed by 55 cycles of 15 s at 95°C and 1 min at 60°C. The relative expression of each mRNA compared with HPRT was calculated according to the equation ΔCt = Ct_target _- Ct_HPRT_. After averaging over at least two technical replicates, the amount of target mRNA was expressed as 2^-(ΔCt)^. In case of time course experiments, the time series of 2^-(ΔCt) ^was normalized to its average value over time. Finally, the averaged value of the indicated number of experiments was calculated for each time point.

### Mathematical model

The reaction network (Figure 3), describing IFNγ signaling in DSL-6A/C1, was translated into a system of ODEs which describe temporal changes of the network components as a function of interactions and transport processes. The model is presented in the Additional file 1.

The kinetic model is based on the following assumptions and simplifications: (a) There is no IFNγ degradation. (b) IFNγ activates Ir and the active receptor IIr phosphorylates STAT1U. (c) The formation of STAT1 D is a rapid and high affinity process [25,26]. (d) Nucleo-cytoplasmic shuttling is modeled as a free diffusion process. (e) The quantification of the confocal microscopy data led to the result that the cytoplasmic area occupied by STAT1 has the same size as the nuclear area (data not shown). (f) Our model includes a reversible binding of STAT1 D to DNA, which determines the resting of STAT1 D in the nucleus [27], (g) Only free nuclear STAT1 D can be dephosphorylated and dissociate into monomers, leading to nuclear STAT1U [26]. (h) Feedback inhibition by SOCS1 may reduce STAT1U phosphorylation [28,29]. (i) The delayed processes of increased STAT1U expression, *SOCS1 *transcription and the negative feedback by SOCS1 are described by a distributed time delay with mean delay times ${\bar{\tau }}_{i}$[30]. The model assumptions are the same as for our model in PSC except of (a) and (b). For further details regarding the assumptions see [10].

### Parameter value estimation

The parameters of the model whose values need to be estimated include reaction constants, delay times, immunoblot scaling factors and initial conditions of some model variables. Their values are estimated by global optimization from protein and mRNA time series for low concentrations (10 ng/ml) and higher concentrations (100 ng/ml) of IFNγ. For estimation we have used a hybrid approach combined of a stochastic simulated annealing algorithm performing a global search and a deterministic trust region algorithm performing a local search. The hybrid approach is implemented in the routine pwFitBoost of the MATLAB toolbox PottersWheel [31]. Results from parameter value estimation are presented in the Additional files 2 and 3.

### Statistical analysis

Results are expressed as means ± standard error of the mean (SEM) for the indicated number of animals or independent cultures per experimental protocol. Statistical significance was determined using tests as specified in the figure legends and where p < 0.05 was considered as statistically significant.

## List of Abbreviations

a.u.: arbitrary unit; ATCC: American Type Culture Collection; BrdU: 5-bromo-2'-deoxyuridine; BSA: bovine serum albumine; CTGF: connective tissue growth factor; DMEM: Dulbecco's Modified Eagle Medium; ERK: extracellular signal-regulated kinase; FCS: fetal calf serum; HPRT: hypoxanthine-guanine phosphoribosyl transferase; IFN: interferon; IMDM: Iscove's modified Dulbecco's medium; IIr: active IFNγ receptor; IL: Interleukin; Ir: inactive IFNγ receptor; PC: pancreatic cancer; PSC: pancreatic stellate cell; PVDF: polivinylidene fluoride; ODE: ordinary differential equation; RSNC: ratio of nuclear versus cytoplasmic concentration of STAT1; SDS: sodium dodecyl sulphate; SEM: standard error of the mean; SOCS: suppressor of cytokine signaling; STAT: signal transducer and activator of transcription; STAT1P: phosphorylated (Phospho-) STAT1; STAT1c: STAT1 in the cytoplasm; STAT1n: STAT1 in the nucleus; STAT1D: phosphorylated STAT1 dimer; STAT1Dc: phosphorylated STAT1 dimer in the cytoplasm; STAT1Dn: phosphorylated STAT1 dimer in the nucleus; STAT1Dnd: phosphorylated STAT1 dimer bound to DNA; STAT1U: unphosphorylated STAT1; STAT1Uc: unphosphorylated STAT1 in the cytoplasm; STAT1Un: unphosphorylated STAT1 in the nucleus; TBS: tris-buffered saline; TGF-β1: transforming growth factor-β1

## Competing interests

The authors declare that they have no competing interests.

## Authors' contributions

FL and BF participated in performing the experiments, data analysis and interpretation of the results. KR established the mathematical model and was involved in the design of the experiments, data analysis and writing of the paper. RP performed the confocal microscopy studies. OW and RJ conceived the study and participated in data analysis and interpretation of the results. RJ also conducted the *in vivo *experiments and wrote the manuscript. All authors have read and approved the final manuscript.

## Supplementary Material

Additional file 1 **Mathematical model describing the reactions of the network in Figure **3. The reaction network was translated into a system of ordinary differential equations (ODE), which describes temporal changes of the network components as a function of interactions and transport processes. The ODE model is shown in the upper part of Additional file 1. The abbreviation *S *stands for STAT1. The variables of the model representing cellular components are concentrations but their units are arbitrary due to the lack of standard curves. The initial conditions of IIr, STAT1Dn and STAT1Dnd were set to zero. They are summarized below the ODE. The initial condition of a variable refers to its value at time point zero and is annotated by (0) after its name. The total concentration of STAT1 and the total IFNγ receptor concentration I are redundant parameters. We have fixed I as 1/10 of initial experimental STAT1. The initial value of STAT1 results from the optimization of the initial values of STAT1Dc, STAT1Uc and STAT1Un. The algebraic equations in the lower part of Additional file 1 relate the model variables to the experimental data. Immunoblot data can be scaled by arbitrary factors. We have chosen different scaling factors for STAT1c, STAT1n, STAT1 D, STAT1Dc, STAT1Dn, annotated by "WB" with the respective form of the protein in the subscript. Scaling factors for STAT1 and *SOCS1 *mRNA have not been included because scaled variables inserted in the model show that the respective scaling factors are redundant parameters. In addition, the ratio of nuclear versus cytoplasmic concentration of STAT1 (RSNC) has been calculated from the confocal microscopy data. Click here for file

Additional file 2 **Estimation of parameter values**. ^a ^While global parameters are independent of the IFNγ concentration, local parameters depend on it. a.u. = arbitrary units. The parameters of the mathematical model include reaction constants and delay times. The parameter values were estimated by global optimization from the protein and mRNA time series. We have used a hybrid algorithm composed of simulated annealing and a local search implemented in the MATLAB toolbox PottersWheel [1]. As a measure for the goodness of how a simulation of the model reproduces experimental data, the following cost function was applied:
$$\chi^{2}(\theta )={\sum_{k=1}^{m}\sum_{l=1}^{d}\left(\frac{y_{kl}^{\exp}-y_{k}^{\mathrm{mod}}}{\sigma_{kl}^{\exp}}\right)}^{2}$$
where θ is the parameter vector, $y_{kl}^{\exp}$ are the experimental data, $y_{k}^{\mathrm{mod}}$ values of observables at time points of the experimental data $\sigma_{kl}^{\exp}$ and is the measurement error of the experimental data [1].
References
1. Maiwald T, Timmer J: **Dynamical modeling and multi-experiment fitting with PottersWheel**. *Bioinformatics *2008, **24:**2037-2043. Click here for file

Additional file 3 **Cost function for different fixed relative concentrations of STAT1Dn for IFNγ = 100 ng/ml**. To estimate an upper boundary for the relative concentration of nuclear STAT1Dn, we fixed the scaling factor WB_STAT1Dn _at different increasing values and re-optimized the other parameter values. The respective values of the cost function are summarized in the left part of the table. For WB_STAT1Dn _= 1000 the cost function reaches a plateau. In the right part of the table the maximal relative concentration of STAT1Dn is calculated from this value. For t = 180 min the concentration of STAT1Dn is 1/500 of the STAT1 concentration and for t = 720 min the concentration of STAT1Dn is 1/1000 of STAT1 concentration. Click here for file
