# Supplementary material for: Studies on mechanisms of interferon-gamma action in pancreatic cancer using a data-driven and model-based approach
Source: Mol Cancer. 2011 Feb 10;10:13. doi: 10.1186/1476-4598-10-13 (PMC3042009; doi:10.1186/1476-4598-10-13)
Supplement: Additional file 1 — Mathematical model describing the reactions of the network in Figure 3. The reaction network was translated into a system of ordinary differential equations (ODE), which describes temporal changes of the network components as a function of interactions and transport processes. The ODE model is shown in the upper part of Additional file 1. The abbreviation S stands for STAT1. The variables of the model representing cellular components are concentrations but their units are arbitrary due to the lack of standard curves. The initial conditions of IIr, STAT1Dn and STAT1Dnd were set to zero. They are summarized below the ODE. The initial condition of a variable refers to its value at time point zero and is annotated by (0) after its name. The total concentration of STAT1 and the total IFNγ receptor concentration I are redundant parameters. We have fixed I as 1/10 of initial experimental STAT1. The initial value of STAT1 results from the optimization of the initial values of STAT1Dc, STAT1Uc and STAT1Un. The algebraic equations in the lower part of Additional file 1 relate the model variables to the experimental data. Immunoblot data can be scaled by arbitrary factors. We have chosen different scaling factors for STAT1c, STAT1n, STAT1 D, STAT1Dc, STAT1Dn, annotated by "WB" with the respective form of the protein in the subscript. Scaling factors for STAT1 and SOCS1 mRNA have not been included because scaled variables inserted in the model show that the respective scaling factors are redundant parameters. In addition, the ratio of nuclear versus cytoplasmic concentration of STAT1 (RSNC) has been calculated from the confocal microscopy data. [file 1476-4598-10-13-S1.DOC]

| **ODE model** |
| --- |
| $\frac{d}{dt}IIr=k_{1}\cdot IFN\gamma\cdot(I-IIr)$ |
| $\frac{d}{dt}SUc=k_{2}\cdot\int_{0}^{\infty} \Gamma_{q_{1}}^{4}(\tau)\cdot IIr(t-\tau)d\tau-k_{3}\cdot IIr\cdot SUc/(1+k_{4}\cdot\int_{0}^{\infty} \Gamma_{q_{2}}^{4}(\tau)SOCS1(t-\tau)d\tau)-k_{5}\cdot SUc+k_{6}\cdot SUn$ |
| $\frac{d}{dt}SDc=k_{3}\cdot IIr\cdot SUc/(1+k_{4}\cdot\int_{0}^{\infty} \Gamma_{q_{2}}^{4}(\tau)SOCS1(t-\tau)d\tau)-k_{7}\cdot SDc$ |
| $\frac{d}{dt}SDn=k_{7}\cdot SDc-k_{8}\cdot SDn+k_{9}\cdot SDnd-k_{10}\cdot SDn$ |
| $\frac{d}{dt}SDnd=k_{8}\cdot SDn-k_{9}\cdot SDnd$ |
| $\frac{d}{dt}SUn=k_{5}\cdot SUc-k_{6}\cdot SUn+k_{10}\cdot SDn$ |
| $\frac{d}{dt}SOCS1=k_{11}+k_{12}\cdot\int_{0}^{\infty} \Gamma_{q_{3}}^{4}(\tau)\cdot SDnd(t-\tau)d\tau-k_{13}\cdot SOCS1$ |
| Kernel of the Gamma function $\Gamma_{q_{i}}^{p}(\tau)=\frac{q_{i}^{p}}{(p-1)!}\tau^{p-1}\cdot e^{-q_{i}\tau}\begin{matrix} \end{matrix}p=4\begin{matrix} \end{matrix}q_{i}=\frac{p}{\overline{\tau_{i}}}$.  The shape is determined by the parameters p and $\overline{\tau_{i}}$ the mean delay time. |
| The parameters *k_i_* are reaction constants. The variables have arbitrary units of concentration. |
| Initial conditions and parameters which are not optimized: $IIr(0)=SDn(0)=SDnd(0)=0\begin{matrix} & \end{matrix}\begin{matrix} & \end{matrix}I=0.08$ Total receptor concentration. |
| **Relations between experimental data (left) and model variables (right)** |
| $STAT1=(SUc+SDc+SDn+SDnd+SUn)/2$ |
| $STAT1c=(SUc+SDc)\cdot WB_{STAT1c}$ |
| $STAT1n=(SDn+SDnd+SUn)\cdot WB_{STAT1n}$ |
| $STAT1D=(SDc+SDn+SDnd)/2\cdot WB_{STAT1D}$ |
| $STAT1Dc=SDc\cdot WB_{STAT1Dc}$ |
| $STAT1Dn=(SDn+SDnd)\cdot WB_{STAT1Dn}$ |
| $SOCS1=SOCS1$ |
| $RSNC=\frac{(SDn+SDnd+SUn)}{\left(SUc+SDc\right)}$ |
| The parameters $WB_{STAT1x}$ are Western blot scaling factors. |
