# Supplementary material for: Studies on mechanisms of interferon-gamma action in pancreatic cancer using a data-driven and model-based approach
Source: Mol Cancer. 2011 Feb 10;10:13. doi: 10.1186/1476-4598-10-13 (PMC3042009; doi:10.1186/1476-4598-10-13)
Supplement: Additional file 2 — Estimation of parameter values. a While global parameters are independent of the IFNγ concentration, local parameters depend on it. a.u. = arbitrary units. The parameters of the mathematical model include reaction constants and delay times. The parameter values were estimated by global optimization from the protein and mRNA time series. We have used a hybrid algorithm composed of simulated annealing and a local search implemented in the MATLAB toolbox PottersWheel [1]. As a measure for the goodness of how a simulation of the model reproduces experimental data, the following cost function was applied: χ2(θ)=∑k=1m∑l=1d(yklexp−ykmodσklexp)2 where θ is the parameter vector, yklexp are the experimental data, ykmod values of observables at time points of the experimental data σklexp and is the measurement error of the experimental data [1]. References 1. Maiwald T, Timmer J: Dynamical modeling and multi-experiment fitting with PottersWheel. Bioinformatics 2008, 24:2037-2043. [file 1476-4598-10-13-S2.DOC]

| **Global parameters and initial conditions ^a^** | | **Local parameters ^a^** |
| --- | --- | --- |
| $\begin{matrix} \text{k}_{1}=0.0009\begin{matrix} \end{matrix}\text{min}^{-1}\text{a.u.}^{-1} \\ \text{k}_{2}=0.069\begin{matrix} \end{matrix}\text{min}^{-1} \\ \text{k}_{3}=0.065\begin{matrix} \end{matrix}\text{min}^{-1}\text{a.u.}^{-1} \\ \text{k}_{4}=0.75\begin{matrix} \end{matrix}\text{a.u.}^{-1} \\ \text{k}_{5}=2.97\begin{matrix} \end{matrix}\text{min}^{-1} \\ \text{k}_{6}=4.13\begin{matrix} \end{matrix}\text{min}^{-1} \\ \text{k}_{7}=0.066\begin{matrix} \end{matrix}\text{min}^{-1} \\ \text{k}_{8}=1.64\begin{matrix} \end{matrix}\text{min}^{-1} \\ \text{k}_{9}=67.63\begin{matrix} \end{matrix}\text{min}^{-1} \\ \text{k}_{10}=309\begin{matrix} \end{matrix}\text{min}^{-1} \end{matrix}$ | $\begin{matrix} \text{k}_{11}=0.01\begin{matrix} \end{matrix}\text{a.u.}\cdot\text{min}^{-1} \\ \text{k}_{12}=211994\begin{matrix} \end{matrix}\text{min}^{-1} \\ \text{k}_{13}=0.063\begin{matrix} \end{matrix}\text{min}^{-1} \\ \overline{\tau}_{1}=275\begin{matrix} \end{matrix}\text{min} \\ \overline{\tau}_{2}=80\begin{matrix} \end{matrix}\text{min} \\ \overline{\tau}_{3}=448\begin{matrix} \end{matrix}\text{min} \\ \text{SUc(0)}=\text{0.95 }\begin{matrix} \end{matrix}\text{a.u.} \\ \text{SDc(0)}=\text{0.003 }\begin{matrix} \end{matrix}\text{ a.u.} \\ \text{SUn(0)}=\text{0.66 }\begin{matrix} \end{matrix}\text{ a.u.} \\ \text{SOCS1(0)}=\text{0.11 }\begin{matrix} \end{matrix}\text{ a.u.} \end{matrix}$ | $\begin{matrix} \text{For IFN}\gamma=100\begin{matrix} \end{matrix}\text{ng/ml:} \\ \text{WB}_{\text{STAT1c}}=0.75 \\ \text{WB}_{\text{STAT1n}}=1.22 \\ \text{WB}_{\text{STAT1D}}=37 \\ \text{WB}_{\text{STAT1Dc}}=21 \\ \text{WB}_{\text{STAT1Dn}}=102538 \\ \\ \text{For IFN}\gamma=10\begin{matrix} \end{matrix}\text{ng/ml:} \\ \text{WB}_{\text{STAT1D}}=49 \\ \\ \end{matrix}$ |
