# Supplementary material for: Studies on mechanisms of interferon-gamma action in pancreatic cancer using a data-driven and model-based approach
Source: Mol Cancer. 2011 Feb 10;10:13. doi: 10.1186/1476-4598-10-13 (PMC3042009; doi:10.1186/1476-4598-10-13)
Supplement: Additional file 3 — Cost function for different fixed relative concentrations of STAT1Dn for IFNγ = 100 ng/ml. To estimate an upper boundary for the relative concentration of nuclear STAT1Dn, we fixed the scaling factor WBSTAT1Dn at different increasing values and re-optimized the other parameter values. The respective values of the cost function are summarized in the left part of the table. For WBSTAT1Dn = 1000 the cost function reaches a plateau. In the right part of the table the maximal relative concentration of STAT1Dn is calculated from this value. For t = 180 min the concentration of STAT1Dn is 1/500 of the STAT1 concentration and for t = 720 min the concentration of STAT1Dn is 1/1000 of STAT1 concentration. [file 1476-4598-10-13-S3.DOC]

| Fixed value for $\text{WB}_{\text{STAT1Dn}}$ | $\chi^{2}$ |  | $\text{WB}_{\text{STAT1Dn}}$=1000 |  |  |
| --- | --- | --- | --- | --- | --- |
| 10 | 2.34 |  | Time [min] | 180 | 720 |
| 100 | 2.14 |  | STAT1Dn [a.u.] | 1.7 | 1.9 |
| 1000 | 2.07 |  | STAT1 [a.u.] | 0.8 | 2 |
| 10000 | 2.07 |  | $\frac{STAT1Dn}{WB_{STAT1Dn}}\cdot\frac{1}{STAT1}$ | 0.002 | 0.001 |
| 100000 | 2.06 |  |
